# Self-Restructuring of Polyhydromethylsiloxanes by the Hydride Transfer Process: A New Approach to the Cross-Linking of Polysiloxanes and to the Fabrication of Thin Polysiloxane Coatings

**DOI:** 10.3390/ma15196981

**Published:** 2022-10-08

**Authors:** Urszula Mizerska, Slawomir Rubinsztajn, Julian Chojnowski, Marek Cypryk, Pawel Uznanski, Agnieszka Walkiewicz-Pietrzykowska, Witold Fortuniak

**Affiliations:** Centre of Molecular and Macromolecular Studies, Polish Academy of Sciences, Sienkiewicza 112, 90-363 Lodz, Poland

**Keywords:** tris(pentafluorophenyl)borane, polyhydromethylsiloxane, hydride transfer polymerization, thin films of silicone, hyperbranched polysiloxanes, cross-linking of polysiloxanes

## Abstract

The branching and cross-linking of siloxane polymers are important processes in silicone technology. A new type of such a process has been developed, which is a self-restructuring of linear polyhydromethylsiloxane (PHMS). This process involves the reorganization of the PHMS to form a highly branched siloxane polymer or finally a cross-linked siloxane network. It occurs through the transfer of a hydride ion between silicon atoms catalyzed by tris(pentafluoromethyl)borane. Its advantage over existing branching and cross-linking reactions is that it runs at room temperature without a low-molecular-weight cross-linker in the absence of water, silanol groups, or other protic compounds and it does not use metal catalysts. The study of this process was carried out in toluene solution. Its course was followed by ^1^H NMR, ^29^Si NMR and FTIR, SEC, and gas chromatography. A general mechanism of this new self-restructuring process supported by quantum calculations is proposed. It has been shown that a linear PHMS self-restructured to a highly branched polymer can serve as a pure methylsiloxane film precursor.

## 1. Introduction

The cross-linking of polysiloxanes involves reactions that link siloxane chains to ultimately form their networks. Usually, it is preceded by branching of the polymer. These two processes are very important in silicone technology [1,2,3,4,5]. There are many methods for the formation of a cross-linked siloxane network. One approach is based on the polymerization or polycondensation of polyfunctional monomers [6,7,8]. Other processes use the cross-linking of linear polysiloxanes with reactive side or end groups which, with limited functional group conversion, result in a branched, soluble, and processable polymer, and their further conversion results in an insoluble polymer network [5,9]. These processes are commonly used in the manufacturing of silicone rubber [9,10,11], silicone coatings [12,13], silicone adhesives and sealants [10,14], silicone precursors of ceramics [15,16], and silicon resins [9,17]. The cross-linking of siloxane polymers can be achieved by three general chemical reactions: condensation, addition to double bonds, and free radical cross-linking [1,2,5]. The condensation reaction requires the presence of a linear polymer functionalized with silanol or another hydrolyzable group and a multifunctional cross-linking agent containing reactive groups such as alkoxysilane, acetoxysilane, oxime, or others [1,2,3,5]. In most cases, various tin or titanium compounds are required to promote condensation chemistry [1,3]. The addition technology is based on the hydrosilylation reaction of silicone oligomers and polymers containing vinyl or allyl groups with Si-H functional cross-linkers [1,3,18]. This reaction requires catalysis via expensive metal complexes such as the Karstedt platinum complex [12,18] or various organometallic complexes of iridium [19], rhodium [20], or platinum II [21]. The silicone networks obtained as a result of these reactions are contaminated with metals, which lowers their thermal, dielectric, and optical properties. New condensation and addition methods for cross-linking polysiloxanes have recently been introduced, but they require laborious syntheses [13,22,23]. Silicone polymers can also be cross-linked by free radicals formed at high temperatures from various azo or peroxide initiators [1,2,24]. The above-mentioned cross-linking methods suffer from several disadvantages, such as the formation of a by-product or catalyst residues that are difficult to completely remove from the cross-linked material, an expensive platinum catalyst, or a high processing temperature. 

In this study, we propose a new approach to creating a cross-linked polysiloxane network from linear polysiloxane. We have found that an inexpensive and easily commercially available linear polyhydromethylsiloxane (PHMS) self-restructures at room temperature. This process involves hydride transfer between silicon atoms in the presence of catalytic amounts of tris(pentafluorophenyl)borane (TPFPB). This restructuring performed in a hydrocarbon solvent such as toluene allows for the conversion of a relatively low-molecular-weight linear PHMS of less than 10,000 into a soluble, highly branched polysiloxane with a molecular weight in the range 10^5^–10^6^. This highly branched polymer is capable of being further transformed into an insoluble, highly cross-linked polymer. This cross-linking process forms a pure methyl siloxane network as it takes place without any low-molecular-weight cross-linker and does not require any organic functional groups. It occurs in the absence of water, silanol, and other protic species. After curing, the catalyst may be deactivated at a moderately elevated temperature [25]. 

Our main goal was to thoroughly investigate the restructuring process of PHMS. The presented research aimed to explain its general course, identifying its component reactions, characterizing the resulting polymer, and identifying its by-products. An additional aim of this study was to show that restructured PHMS is a promising candidate to produce thin, fully cross-linked siloxane coatings that are free of heavy metals.

The preliminary knowledge on PHMS restructuring comes from the discovery of the hydride transfer ring opening polymerization (HTROP) of 1,3,5,7-tetrahydrotetramethyl- cyclotetrasiloxane (D^H^_4_) [26]. This polymerization is catalyzed by TPFPB and occurs by silicon-to-silicon hydride transfer with the opening of siloxane bonds of the cyclic monomer and adding it to the growing polymer chain by forming a new siloxane bond. This polymerization is accompanied by branching of the polymer formed, which occurs via hydride transfer according to general Equation (1). The branching units are produced at the expense of the evolution of volatile methylsilane. It can be assumed that like the HTROP of D^H^_4_, the formation of a branched siloxane unit in the self-restructuring of linear PHMS can be represented by the same general Equation (1) and follows a similar hydride transfer pathway.

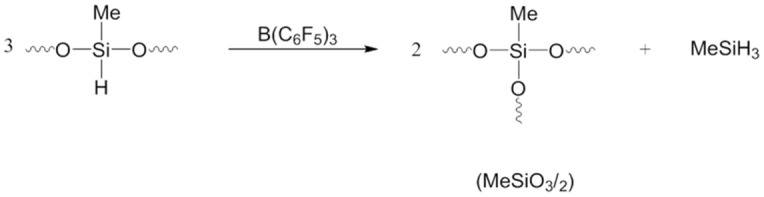
(1)

## 2. Experimental Section

### 2.1. Materials

Linear polyhydromethylsiloxane PHMS with trimethylsiloxane chain ends was the product of ABCR with a declared viscosity of 15–25 cSt. It had molecular mass of about 2600 Da, as confirmed via ^1^H NMR and ^29^Si NMR. Tris(pentafluorophenyl)borane (TPFPB), a product of TCI (Tokyo Chemical Industry, Tokyo, Japan) of declared purity >98%, was purified by sublimation under reduced pressure at 90 °C. The catalyst purity was checked via ^19^F NMR. A stock solution of the catalyst was prepared by dissolving 0.97 g (0.00189 mol) of TPFPB in 5 mL of dry toluene in a small Schlenk ampule under dry nitrogen. 

### 2.2. Analytical Methods

The ^1^H NMR, ^29^Si NMR, and ^19^F NMR spectra in C_6_D_6_ were obtained with the Avance NEO 400 NMR spectrometer, operating at 400.15 MHz for ^1^H and 376.55 MHz for ^19^F and 79.50 MHz for ^29^Si, which consists of a narrow bore 9.4 Tesla Ascend superconducting magnet and an Avance NEO console from Bruker Corporation (Karlsruhe, Germany) operating with a Sample Case Plus autosampler. The spectrometer is equipped with 5 mm high resolution dual channel ^2^H/^1^H(^19^F)/BB i-Probe (Bruker, Karlsruhe, Germany) with a z-gradient coil capable of tuning to nuclei ^19^F/^31^P^199^Hg and ^17^O^109^Ag on the BB channel with an automatic tuning and matching system (ATMA, Bruker, Karlsruhe, Germany). The spectrometer uses a BCU-I, controlled by a Bruker Smart Variable Temperature (BSVT) system, for temperature regulation and stabilization. The spectrometer is operated using the TopSpin 4.1 program (Bruker, Karlsruhe, Germany). The Inverse Gated Decoupling technique was used to quantitatively acquire the ^29^Si NMR spectra. 

Fourier-transform Infrared Spectroscopy (FTIR) spectra were recorded using a Nicolet 380 spectrophotometer (Thermo Scientific, Waltham, MA, USA). The analyzed sample was placed in a cuvette with ZnSe windows and 1 mm thick Teflon spacers.

Gas chromatography analysis was performed using a Hewlett Packard 6890 II apparatus equipped with a TCD detector (Agilent Technologies, Santa Clara, CA, USA) and an HP-1 column (30 m × 0.53 mm × 1 μm). The injector and detector temperature was 250 °C. The carrier gas was helium. Temperature program: the column was kept at 60 °C for 4 min, then heated to 240 °C at a rate of 5 °C/min.

Differential scanning calorimetry (DSC) was performed by heating the sample from −50 °C to 350 °C with a rate of 10 °C/min using DSC 2920 (TA Instruments, New Castle, PA, USA).

Thermogravimetric analysis (TGA) was performed by heating the sample under nitrogen from 25 °C to 1000 °C with a rate of 5 °C/min using a TGA 5500 analyzer (TA Instruments, New Castle, PA, USA).

Size exclusion chromatography (SEC) was performed in toluene at 30 °C using PSS SDV Analytical 500 Å + 10,000 Å columns. The SEC system was equipped with miniDAWN TREOS Multi-Angle Light Scattering for Absolute Macromolecular Analysis (Wyatt Technology Europe, Dernbach, Germany) and a Knauer Refractive Index detector. 

### 2.3. Restructuring of PHMS 

All operations in the preparation of the reaction mixture were carried out under an atmosphere of dried nitrogen. In a typical experiment, a solution of 4 g of PHMS dissolved in 18 mL of dried toluene was placed in a flask purged with nitrogen, equipped with a magnetic stirrer, a nitrogen gas inlet, and a gaseous products outlet through a bubbler. The solution of tris(pentafluorophenyl)borane (1.9 × 10^−4^ mol) dissolved in 0.5 mL of dried toluene was introduced to the stirred solution of PHMS at 30 °C by means of a syringe through a septum. Samples were withdrawn at the desired time intervals by means of a hypodermal syringe through a septum, neutralized by the addition of 4-ethylpyridine, and were subjected to analysis via ^1^H NMR, ^29^Si NMR, FTIR, and GCMS. The polymeric product was precipitated from the toluene solution upon the addition of methanol and was analyzed via ^1^H NMR, ^29^Si NMR, FTIR, and SEC. 

### 2.4. Preparation and Characteristics of Thin Films

The toluene solutions containing from 10 to 20 wt% of the highly branched siloxane polymer were used to prepare thin coatings with a thickness of about 1 μm. The siloxane films were prepared on a silicon wafer with a diameter of 5 cm using a BIDTEC SP 100 spincoater at 3 successive speeds: 300 (for the first 6 s of spreading the solution), 1000 (for the first 6 s of the film’s creation), and 3000 (for the last 6 s of drying the film) rpm. Each sample was deposited from a 100 μL solution in toluene. The films formed were solid and not sticky after toluene evaporation. They were allowed to cure in ambient conditions in air overnight at RT and subsequently heated up at 150 °C for 90 min. The conversion of the residual SiH groups was followed by FTIR spectroscopy. The solid films were removed from the wafer and analyzed via DSC and TGA.

### 2.5. Theoretical Calculations

All quantum mechanical calculations were performed using the Gaussian 16 suite of programs [27]. The geometries of the reagents and complexes were optimized using the hybrid B3LYP density functional [28] corrected for dispersion interactions using the Grimme GD3 empirical term [29], with the Def2-SVP basis set [30] in the gas phase. All stationary points were identified as stable minima by frequency calculations. The vibrational analysis provided thermal enthalpy and entropy corrections at 298 K within the rigid rotor/harmonic oscillator/ideal gas approximation [27]. Thermochemical corrections were scaled by a factor of 0.99 [31]. More accurate single-point electronic energies were obtained using the B3LYP functional, including Grimme GD3 dispersion correction, with the larger Def2-TZVP basis set for the Def2-SVP optimized geometries. This level of theory is denoted as B3LYP-GD3/Def2TZVP//Def2SVP. The integration grid was set to ultrafine.

## 3. Results and Discussion

### 3.1. Study of Restructuring Process

**Figure 1:**
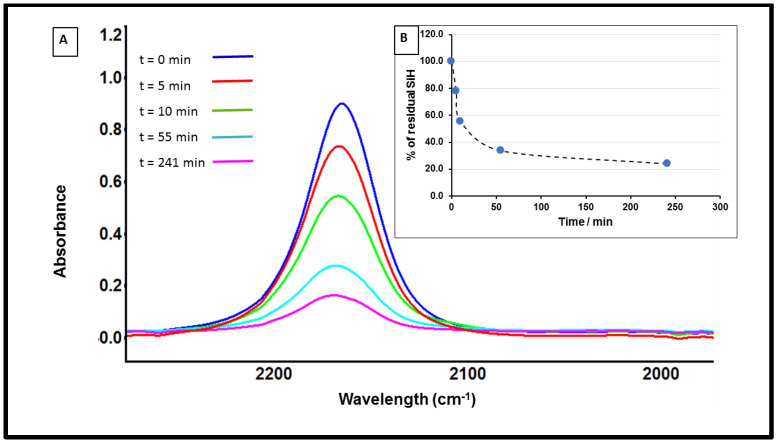
(**A**)—IR spectra of SiH band taken during reaction of 20 wt% PHMS solution in toluene at 30 °C in the presence of 1 × 10^−2^ mol/L of B(C_6_F_5_)_3_. (**B**)—SiH conversion vs. time dependence of the 20 wt% PHMS restructuration at the same conditions.

A series of chain restructuring reactions of linear PHMS catalyzed by B(C_6_F_5_)_3_ in toluene was performed using various initial polymer and catalyst concentrations. The reaction was followed by sampling and the analysis of quenched samples via FTIR, ^1^H NMR, ^29^Si NMR, gas chromatography, and SEC. The FTIR spectra showed that the intensity of the SiH band at about 2170 cm^−1^, as expected, strongly decreased during the reaction, as shown in Figure 1. 

**Figure 2:**
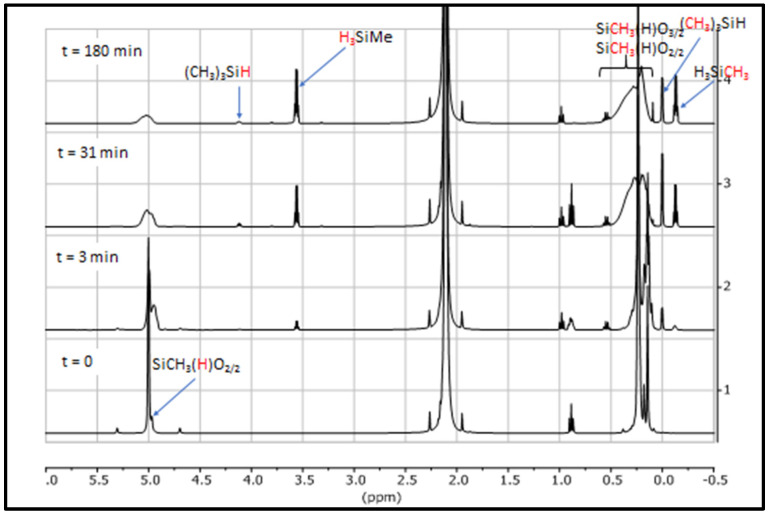
The ^1^H NMR spectra acquired during reaction of 20 wt% PHMS solution in toluene in the presence of 1 × 10^−2^ mol/L of B(C_6_F_5_)_3_ at 30 °C.

**Figure 3:**
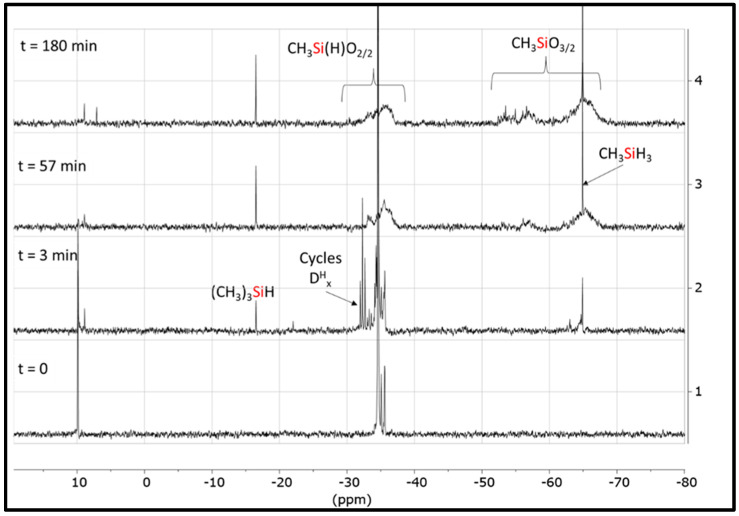
The ^29^Si NMR spectra acquired during the reaction of 20 wt% PHMS solution in toluene in the presence of 1 × 10^−3^ mol/L of B(C_6_F_5_)_3_ at 30 °C.

The signals of CH_3_Si(H)O_2/2_ (D^H^) at 5.0 ppm in ^1^H NMR and CH_3_Si(H)O_2/2_ at −36 ppm in ^29^Si NMR spectra became broader and their integration decreased as the reaction progressed, as shown in Figure 2 and Figure 3, which indicated the consumption of D^H^ units. On the other hand, a broad signal of the CH_3_SiO_3/2_ group (T group) appeared in the ^29^Si NMR spectrum with a maximum at −65 ppm, the intensity of which increased as the reaction progressed, which indicated the formation of T branching units, as shown in Figure 3. The conversion of D^H^ and the formation of T units was also confirmed by the strong broadening of the CH_3_SiO_x_ signal, where x = 2/2 or 3/2, from a narrow peak at 0.24 ppm to a broad signal from 0.05 to 0.6 ppm in ^1^H NMR, as shown in Figure 2.

During this reaction, a gaseous product was released which was trapped in chilled chloroform in a separate experiment. It was subjected to ^1^H NMR and mass spectroscopy analyses, which showed that the gas evolved was composed of the two compounds, MeSiH_3_ and Me_3_SiH (Supporting Information Figure S1). Part of these volatile by-products was dissolved in the reaction system, which was manifested by the appearance of the corresponding signals in the ^1^H NMR spectrum, i.e., a quartet of MeSiH_3_ at 3.58 ppm and a quartet of CH_3_SiH_3_ at −0.1 ppm, a multiplet of (CH_3_)_3_SiH at 4.13 ppm and a doublet of (CH_3_)_3_SiH at 0.01 ppm; see Figure 2 and Figure S1. The corresponding (CH_3_)_3_SiH and MeSiH_3_ sharp signals were also observed in the ^29^Si NMR spectrum at −16.43 and −64.89 ppm, respectively (Figure 3). The chemical identity of these volatile compounds was confirmed via GCMS analysis, as shown in Figure S2A–C.

**Scheme 1:**
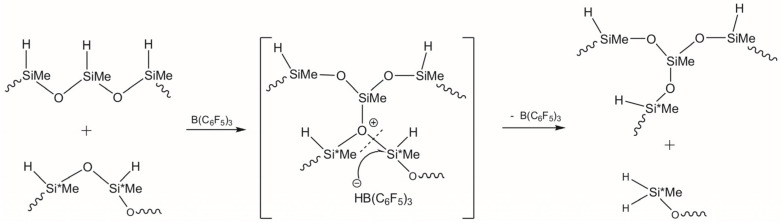
Initial chain branching reaction.

The restructuring process of PHMS includes three separate reactions. Their mechanism involves the hydride transfer process, which can be understood by formally assuming the formation of the tertiary oxonium salt as an intermediate product. TPFPB is the catalyst in these reactions, as it easily takes out the hydride anion from silicon, forming an unstable borate anion which readily gives back the H^−^ to an electrophilic center [32,33]. The first reaction (initial chain branching) is the reaction between two fragments of the polymer chains, shown in Scheme 1. A hydride ion is abstracted from silicon of the one chain fragment by TPFPB and is transferred to a silicon atom of the other fragment with the cleavage of the chain and the formation of an O_1/2_(Me)SiH_2_ group at the formed chain end. The other formed chain end is linked by a new siloxane bond to the silicon from which the hydride ion was taken, thus forming the chain branching unit.

The concentration of the O_1/2_(Me)SiH_2_ end unit in the reaction system is at a very small level, as they are not detected by spectroscopic analysis. This can be explained by the rapid subsequent reactions involving the terminal group O_1/2_(Me)SiH_2_, as discussed below.

**Figure 4:**
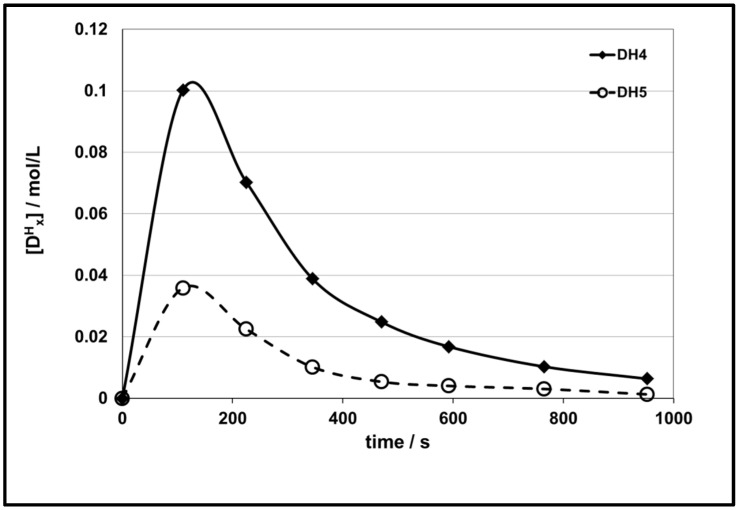
Cyclic oligomers’ formation and disappearance during the restructuring of PHMS in its 20% solution in toluene in the presence of 1 × 10^−2^ mol/L of (C_6_F_5_)_3_B at 30 °C.

**Scheme 2:**
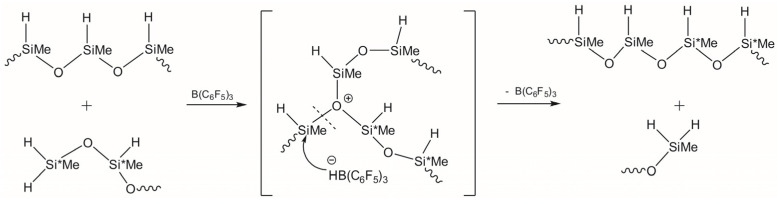
Chain scrambling reaction.

**Scheme 3:**
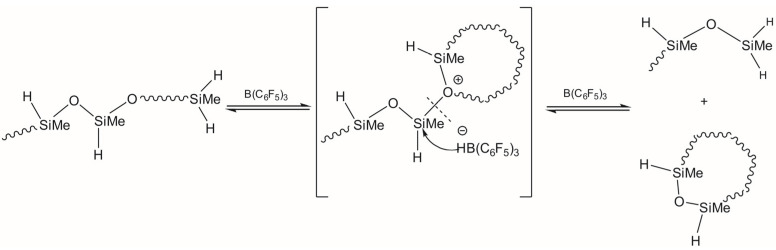
Formation of cyclic oligomers, D^H^_x_, during the restructuring of PHMS.

The second reaction (chain scrambling) is illustrated in Scheme 2. The intermolecular process causes chain reshuffling but does not form any chain branching. However, a competing intramolecular process, the back biting reaction, produces cyclic methylhydrosiloxane oligomers, (OSiMe(H))_x_, mostly tetramer D^H^_4_ and pentamer D^H^_5_ according to Scheme 3. A considerable amount of them is formed at the beginning of the restructuring process, when long segments of D^H^ units are still available. The presence of cyclic species was confirmed via GCMS (Figure S3) and ^29^Si NMR analysis (sharp signals at −32.3 ppm), as shown in Figure 3. They undergo hydride transfer ring opening polymerization [26], which occurs at a higher speed than the restructuring and disappears from the reaction system, as tracked via gas chromatography; Figure 4. As the conversion of D^H^ units proceeds, such intramolecular reactions create other macrocyclic structures built into the polymer macromolecule.

**Scheme 4:**
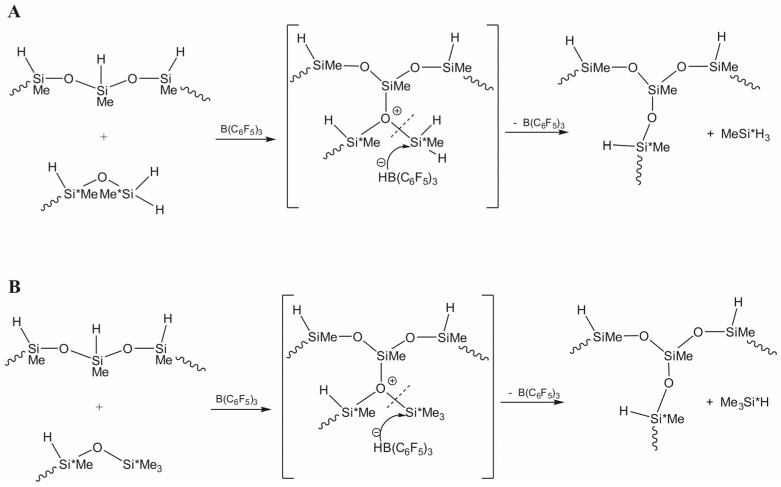
Branching reaction with the release of (**A**) MeSiH_3_ and (**B**) Me_3_SiH.

The third reaction leading to chain branching with the release of MeSiH_3_ is shown in Scheme 4A. It is also a reaction between the O_1/2_(Me)SiH_2_ chain end and the MeHSiO_2/2_ unit inside the chain, but it consists of the formation of an intermediate oxonium salt by the nucleophilic attack of oxygen in the terminal O_1/2_(Me)SiH_2_ group on the activated silicon center inside the chain. The hydride ion abstracted from this silicon by borane is subsequently transferred to the silicon of the O_1/2_(Me)SiH_2_ end unit to form volatile MeSiH_3_ by the rupture of the Si-O bond. The remaining chain end links to the silicon by the new Si-O bond, resulting in the generation of a chain branching unit.

The nucleophilic attack of oxygen in the terminal -OSiMe_3_ group on the activated silicon center creates an analogous oxonium intermediate salt which then undergoes transformation to generate Me_3_SiH and new chain branching, as shown in Scheme 4B. Branching units are formed in the first and third reaction. All three steps may occur intra- and intermolecularly. The by-products, MeSiH_3_ and Me_3_SiH, mentioned above are volatile gases with a boiling point of −57.5 °C and 6.7 °C, respectively. They can be easily removed from the polymer product during the evaporation of the solvent. Due to their flammability, appropriate precautions should be taken when the reaction is carried out on a large scale. 

**Scheme 5:**
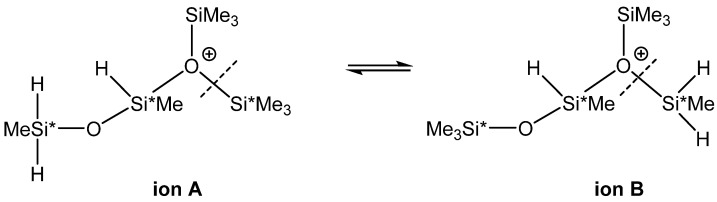
The SiMe_3_ group migration in oxonium ion between oxygen atoms of MeH_2_SiOSiHMeOSiMe_3_.

Preliminary quantum chemical calculations of the thermodynamics of the restructuring reaction and of the formation of some complexes of TPFPB with silyl hydrides and the generation of some trisilyloxonium ions were carried out. The results of these studies are gathered in Table 1. They support the PHMS restructuring mechanism and provide a closer understanding of the process. The thermodynamics results for model reactions corresponding to Scheme 1 and Scheme 4A, entries 1 and 2 in Table 1, respectively, clearly show that the generation of the branching units, according to these reactions, are thermodynamically favored. The replacement of hydrogen by a methyl group makes the complex formation of borane with Me_n_SiH_4-n_, n = 1–3, compounds series easier, which is not only marked by the increase in the energy of the SiH-TPFPB complex formation, but also by the decrease in the hydrogen–boron distance (Table 1, entries 3–5). An even stronger SiH-TPFPB complex was formed when hydrogen was replaced with a trimethylsiloxy group (Table 1, entry 6). This confirms the poor ability of MeSiH_3_ to act as a hydride donor. Consequently, the formation of the MeSiH_3_-TPFPB complex is unlikely, and therefore, the reverse reaction of MeSiH_3_ leading to the formation of the O_1/2_MeSiH_2_ end group is slow or practically does not occur. The formation of the trisilyloxonium salt produced from the O_1/2_Si(Me)H_2_ chain fragment and Me_3_SiH requires less energy input and less entropy loss as compared with that from the O_1/2_SiMe_3_ fragment, as shown in Entry 7, which presents the enthalpy and free energy of the direct isomerization of ion A to ion B (Scheme 5). The results of these calculations indicate that the oxygen of the O_1/2_Si(Me)H_2_ fragment is considerably more nucleophilic than the corresponding oxygen of the OSiMe_3_ group and explains why the O_1/2_MeSiH_2_ end group is not detected in the PHMS restructuring system via spectroscopic methods.

**Figure 5:**
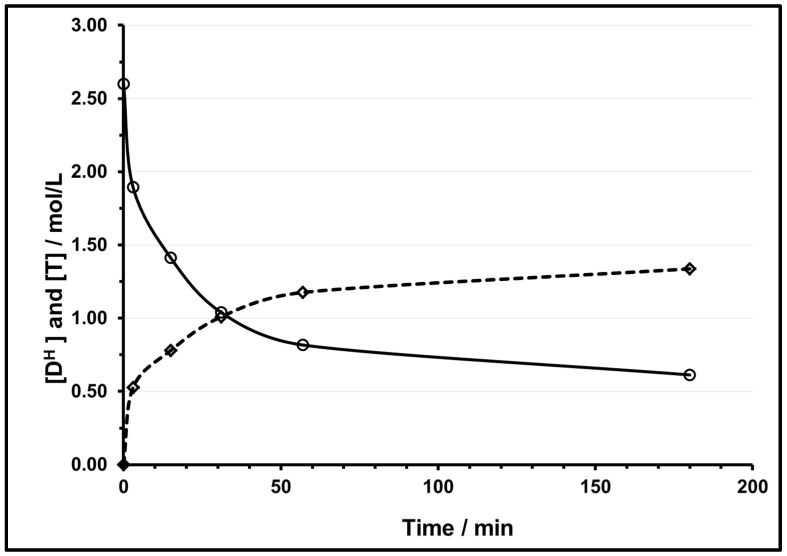
HMeSiO_2/2_ (D^H^) unit conversion vs. time (-) and chain branching units (MeSiO_3/2_) (T) formation vs. time (---) dependences in the restructuration of 20 wt% PHMS solution in toluene in the presence of 7.6 × 10^−3^ mol/L of B(C_6_F_5_)_3_ at 30 °C.

The comparison of the rate of the formation of the branching T units with the rate of the disappearance of D^H^ units calculated from the ^1^H NMR spectra of the reaction mixture is shown in Figure 5. On average, the ratio of the D^H^ unit’s disappearance to the formation of T branching units is 3/2, confirming that the linear PHMS restructuring process follows a similar general mechanism as the hydride transfer polymerization HTROP of D^H^_4_, shown in Equation (1) [26].

**Figure 6:**
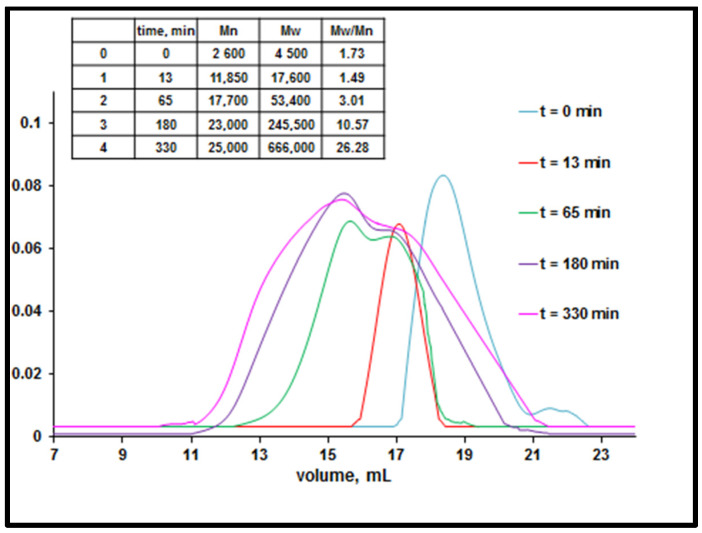
The variation in the molar mass distribution of the PHMS polymer during the restructuring in 20 wt% toluene solution at 30 °C in the presence of 1 × 10^−2^ mol/L TPFPB.

PHMS restructuring followed by FTIR and NMR spectroscopies slows down strongly with high D^H^ unit conversion, as shown in Figure 1 and Figure 5. The reasons for this are a strong increase in molecular weight, shown in Figure 6 and also in Supporting Information (Figure S4) and extensive branching, reducing the mobility of polymer chains, which was also manifested by an increase in the viscosity of the polymer solution. Increasing the concentration of PHMS causes not only an increase in the reaction rate, but also earlier gelling of the reaction mixture due to the higher rate of intermolecular processes that lead to cross-linking, as shown in Table 2. It was found that no gelation of the reaction mixture was observed despite the high conversion of D^H^ units when the initial PHMS concentration was below 10%. This discovery is especially important when the restructured polymer solution is used as a precursor for the formation of siloxane films as it significantly increases its operating window, as discussed in the next section of this manuscript.

**Scheme 6:**
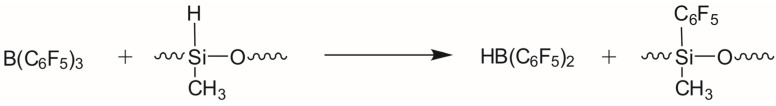
Reaction of the pentafluorophenyl group exchange for hydrogen in the SiH group in the polymer.

Another reason for slowing down the restructuring process is that the borane catalyst is not stable in this system, as it undergoes a slow reaction of the pentafluorophenyl group exchange for hydrogen with the SiH group in the polymer, as shown in Scheme 6 [25]. The formed HB(C_6_F_5_)_2_ is not an active catalyst for the HTROP reaction. The representative ^19^F NMR spectra confirming the decomposition of TPFPB are shown in the Supplemental Information (Figure S5).

Restructuring leads to a high molecular weight of the branched polymer, although the initial molecular weight of PHMS is low. Its increase during the reaction is shown in Figure 6. The results of PHMS restructuring are summarized in Table 2.

The restructuring reaction produces a high-molecular-weight, densely branched, soluble polymer with a compact structure. It has reactive SiH groups that can be used for further functionalization or for the formation of an insoluble cross-linked polymer. It is suitable for use as reactive blocks for the creation of hybrid materials or for the generation of thin films.

### 3.2. Preparation and Characteristics of Thin Films

The ability of restructured siloxane polymers to produce cross-linked film was demonstrated by generating thin films via spin coating on silicon wafers using 10 wt% and 20 wt% toluene solutions of the restructured PHMS. The polymers used and the characteristics of the produced films are shown in Table 3. The 10 wt% PHMS in toluene, which was reacted for only 40 min in the presence of TPFPB, did not form a continuous smooth film (SI Figure S6A). The formation of a poor-quality film can be explained by the relatively low molecular weight of the branched polymer, shown in Figure 6. The same solution, which was reacted for an additional 2.5 h and 23 h, formed a good-quality continuous film with a thickness of 500 nm to 1 micron (SI Figure S6B). A good-quality film was also obtained from a 20 wt% PHMS solution, which was reacted for 2 h. However, the same solution gelled after about 8 h of reaction and could not be used to produce good-quality films via spin coating. The produced films were solid and non-sticky, suggesting that their glass transition temperature was above room temperature. Films were exposed to air at room temperature for 24 h. The content of the SiH groups in the formed film was monitored via infrared spectroscopy, as shown in Figure S7. The rate of SiH conversion in the solid film was much slower than in solution, which can be explained by the lower molecular mobility of the restructured, highly branched macromolecule the after removal of the solvent. The hydrolysis of SiH groups via ambient water and SiH + SiOH condensation catalyzed by borane [34,35,36] may also contribute to the removal of SiH groups. 

**Figure 7:**
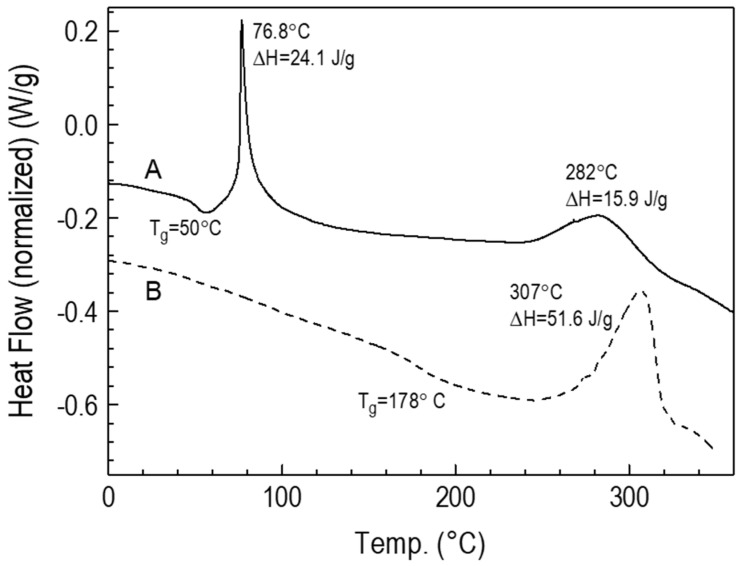
**A**—DSC curve of thin film #4 after being held 24 h at RT; **B**—DSC curve of thin film #4 90 min post-bake at 150 °C in air.

**Figure 8:**
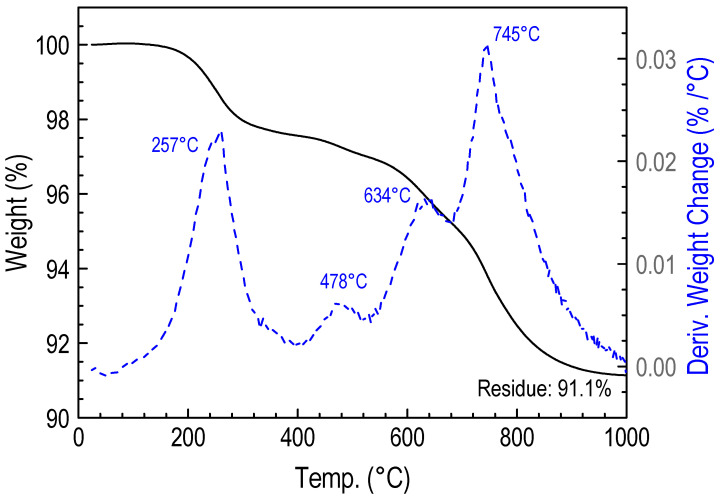
TGA curve of the thin film #4 90 min post-bake at 150 °C in air.

DSC analysis of the films kept at room temperature in air was performed under a nitrogen atmosphere. A representative DSC curve of Film 4 is shown in Figure 7A. The produced film has a glass transition temperature of 50 °C, which additionally confirms that the molecular mobility of the restructuring polymer is frozen due to its glassy state at room temperature. Two exothermic peaks with onset temperatures at 75 °C and 252 °C were detected. The 75 °C exothermic peak resulted from further TPFPB catalyzed cross-linking reactions, i.e., O_2/2_(Me)SiH restructuring and SiH + SiOH condensation, the speed of which increased as a result of the loosening of the polymer structure and the greater mobility of the macromolecular segments above the glass temperature. This relationship between the cross-linking rate, the glass transition temperature of the polymer matrix, and the curing temperature is well known in the field of thermosetting resin technology [37,38]. On the basis of these DSC results, an additional increase in the conversion of SiH groups and a corresponding increase in the glass transition temperature to 178 °C was obtained by subsequent baking of the thin film at 150 °C for 90 min in air, as shown in Figure 7B. The second exothermic peak (Curve A) with an onset at 252 °C and the exothermic peak (Curve B) with an onset at 272 °C can be attributed to the cleavage of the Si-H bond by SiOH with the release of hydrogen, which takes place above 200 °C [39]. An alternative route for thermal cross-linking via the oxidation of the Si-H bond and subsequent condensation with the release of water, as recently proposed by Brook et al. for PHMS [40], was less likely in this case because the dry nitrogen atmosphere was used in the DSC experiment. The post-baked film showed high thermal stability with only 2.5% weight loss below 400 °C and a high ceramic yield of 91% at 1000 °C under nitrogen, as shown in Figure 8.

## 4. Conclusions

The restructuring of linear polyhydromethylsiloxane occurs through silicon-to-silicon hydride ion transfer mediated by tris(pentafluorophenyl)borane, leading to the formation of branching siloxane units. This reaction converts the linear polysiloxane into a highly branched and ultimately cross-linked polymer. It was demonstrated that from a relatively short-chain linear polysiloxane having on average 40 siloxane units, a soluble, high-molecular-weight polymer of 10^5^ Da could be obtained. This highly branched polymer was then converted to a pure cross-linked methyl siloxane network. This reaction could be used to produce thin silicone coatings from readily available and inexpensive input materials. 

## Figures and Tables

**Table 1:** Thermodynamic functions and hydrogen–boron bond length of Me_n_SiH_3-n_, n = 1–3, complexes with TPFBP and thermodynamic functions of the formation of some siloxonium ions.

No.	Reaction	ΔH^298^ Kcal/mol	ΔG^298^ Kcal/mol	r(H-B) Å
1	2 Me_3_SiOSiHMeOSiMe_3_ → Me_3_SiOSiMeH_2_ + (Me_3_SiO)_3_SiMe	−3.2	−5.3	
2	Me_3_SiOSiHMeOSiMe_3_ + Me_3_SiOSiMeH_2_ → MeSiH_3_ + (Me_3_SiO)_3_SiMe	−10.1	−7.4	
3	Me_3_SiH + B(C_6_F_5_)_3_ → Me_3_SiH-B(C_6_F_5_)_3_	−8.5	4.0	1.516
4	Me_2_SiH_2_ + B(C_6_F_5_)_3_ → Me_2_SiH_2_-B(C_6_F_5_)_3_	−5.6	4.9	2.302
5	MeSiH_3_ + B(C_6_F_5_)_3_ → MeSiH_3_-B(C_6_F_5_)_3_	−3.9	5.7	2.540
6	Me_3_SiOSiHMeOSiMe_3_ + B(C_6_F_5_)_3_ → (Me_3_SiO)_2_SiMeH-B(C_6_F_5_)_3_	−11.3	4.7	1.525
7	(Me_3_Si)_2_O^(+)^(SiHMeOSiMeH_2_) (A) → (Me_3_Si)(MeH_2_Si)O^(+)^(SiHMeOSiMe_3_) (B)	−2.4	−3.2	

**Table 2:** Restructuring of PHMS in toluene solution at 30 °C catalyzed by 1 × 10^−2^ mol/L B(C_6_F_5_)_3_.

Experiment Number	Initial PHMS Concentration w%	[D^H^] mol/L	Time to Gelation Min	D^H^ Conversion at Gelation %
1	10	1.3	No Gel	No Gel
2	20	2.6	260	76
3	30	4.0	6	60

**Table 3:** Fabrication of thin films from restructured high-molecular-weight polymers.

Film #	Polymer Concentration/wt%	Time of Reaction/h	Initial % HMeSiO Conversion	Film Quality	% HMeSiO Conversion after 24 h @ RT	% HMeSiO Conversion after Postbake @150 °C
1	10	0.67	56.7	Poor	71.9	83.7
2	10	3	72.6	Good	77.4	88.5
3	10	24	82	Good	ND	86
4	20	2	70.4	Good	77	87.8
5	20	8	77	Gel/Poor	ND	ND

## Data Availability

The data presented in this study are available on request from the corresponding author.

## Supplementary Materials

The following supporting information can be downloaded at: https://www.mdpi.com/article/10.3390/ma15196981/s1, Figure S1. The ^1^H NMR spectrum of the volatile products of the reaction 20 wt% PHMS solution in toluene in the presence of 1 × 10^−2^ mol/L of B(C_6_F_5_)_3_ at 30 °C. The trapping of gaseous products was not quantitative as a considerable part of highly volatile MeSiH_3_ was not dissolved in cold chloroform. Figure S2. GCMS chromatogram of the volatile products of reaction mixture of 20 wt% PHMS solution in toluene in the presence of 1 × 10^−2^ mol/L of B(C_6_F_5_)_3_ at 30 °C. Figure S3. GCMS chromatogram of the reaction mixture of 20 wt% PHMS solution in toluene in the presence of 1 × 10^−2^ mol/L of B(C_6_F_5_)_3_ at 30 °C. Figure S4. The variation in the molar mass distribution of the PHMS polymer during the restructuring in 20 wt% toluene solution at 30 °C in the presence of 1 × 10^−2^ mol/L of TPFPB obtained via Multi-Angle Light Scattering detector. Figure S5. The ^19^F NMR spectra obtained during the reaction of 20 wt% PHMS solution in toluene in the presence of 1 × 10^−2^ mol/L of B(C_6_F_5_)_3_ at 30 °C. Figure S6. (A)—Image of the poor-quality film (Table 3, Film #1); (B)—Image of the good-quality film (Table 3, Film #2). Figure S7. (A)—IR spectra of the solid film #2 held at ambient conditions for 24 h. (B)—SiH conversion vs. time of holding the solid film #2 in ambient conditions and 90 min post-bake at 150 °C.
