# Supplementary material for: Self-Restructuring of Polyhydromethylsiloxanes by the Hydride Transfer Process: A New Approach to the Cross-Linking of Polysiloxanes and to the Fabrication of Thin Polysiloxane Coatings
Source: Materials (Basel). 2022 Oct 8;15(19):6981. doi: 10.3390/ma15196981 (PMC9570814; doi:10.3390/ma15196981)
Supplement: Supplementary file 1 [file materials-15-06981-s001.zip › materials-1933189-supplementary.pdf]

Supplementary Information

Self-restructuring of polyhydromethylsiloxanes by the hydride transfer process. New approach to the cross-linking of polysiloxanes and to the fabrication of thin polysiloxane coatings

Urszula Mizerska, Slawomir Rubinsztajn, Julian Chojnowski, Marek Cypryk, Pawel Uznanski, Agnieszka Walkiewicz-Pietrzykowska and Witold Fortuniak*

Centre of Molecular and Macromolecular Studies, Polish Academy of Sciences, Sienkiewicza 112, 90-363 Lodz, Poland

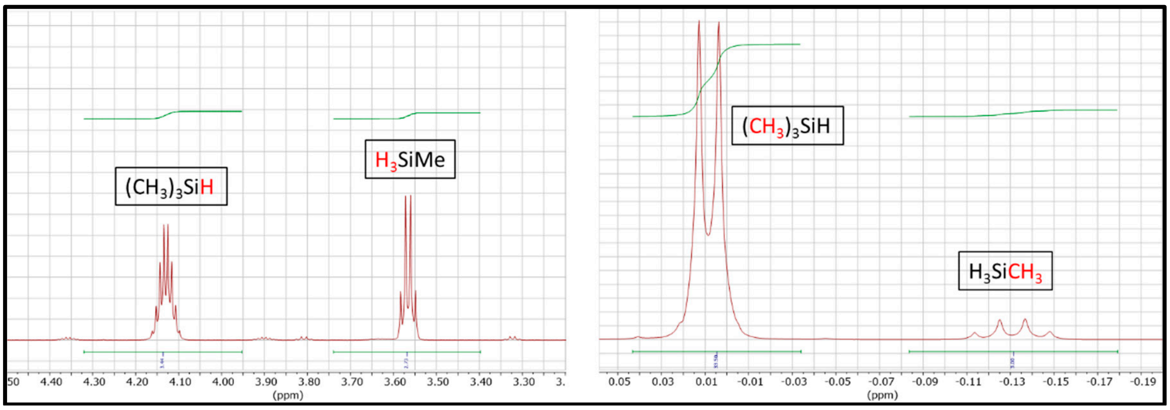

Figure S1. ^{1}H NMR spectrum of the volatile products of the reaction 20 wt % PHMS solution in toluene in the presence of 1x 10^{-2} mol/L of $B(C_6F_5)_3$ at 30 °C **.** The trapping of gaseous products was not quantitative as a considerable part of highly volatile MeSiH3 was not dissolved in cold chloroform.

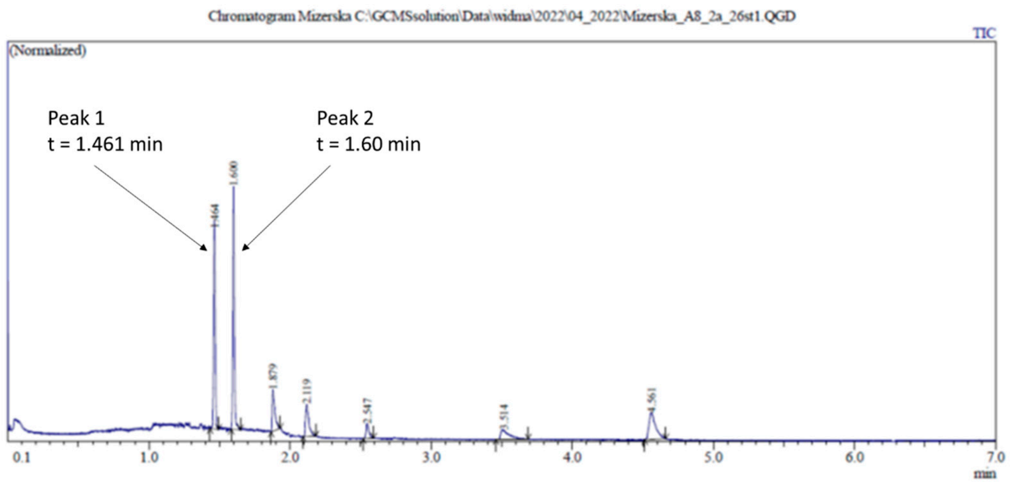

Figure S2A. GCMS chromatogram of the volatile products of reaction mixture of 20 wt % PHMS solution in toluene in the presence of 1 10^{-2} mol/L of $B(C_6F_5)_3$ at 30 °C

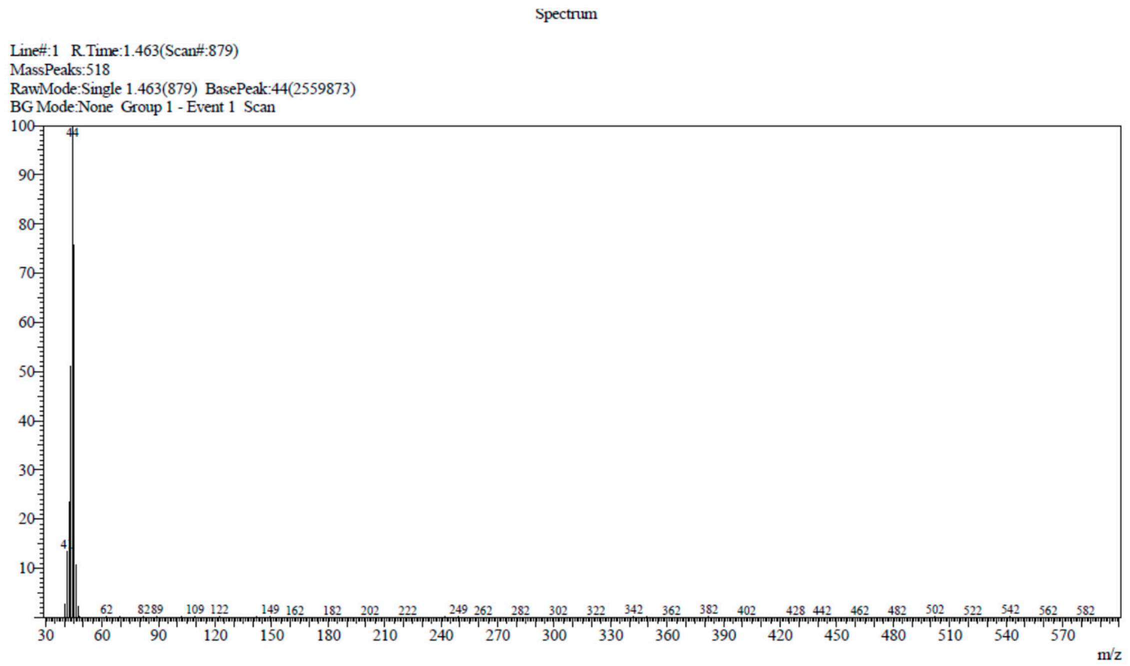

Figure S2B. MS analysis of the peak 1.

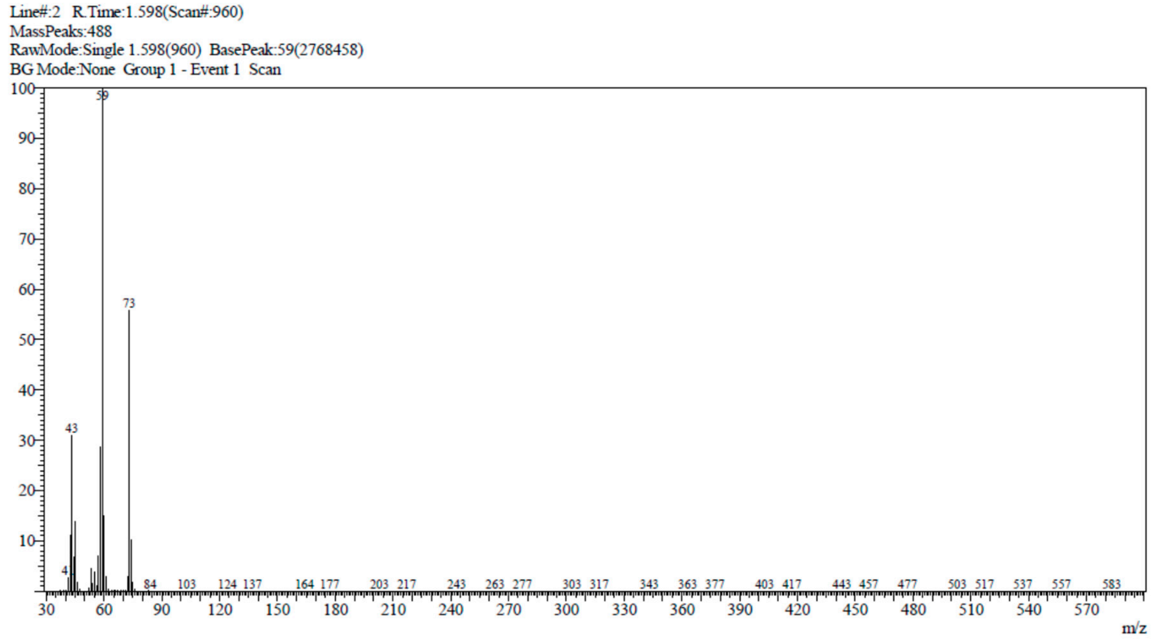

Figure S2C. MS analysis of the peak 2.

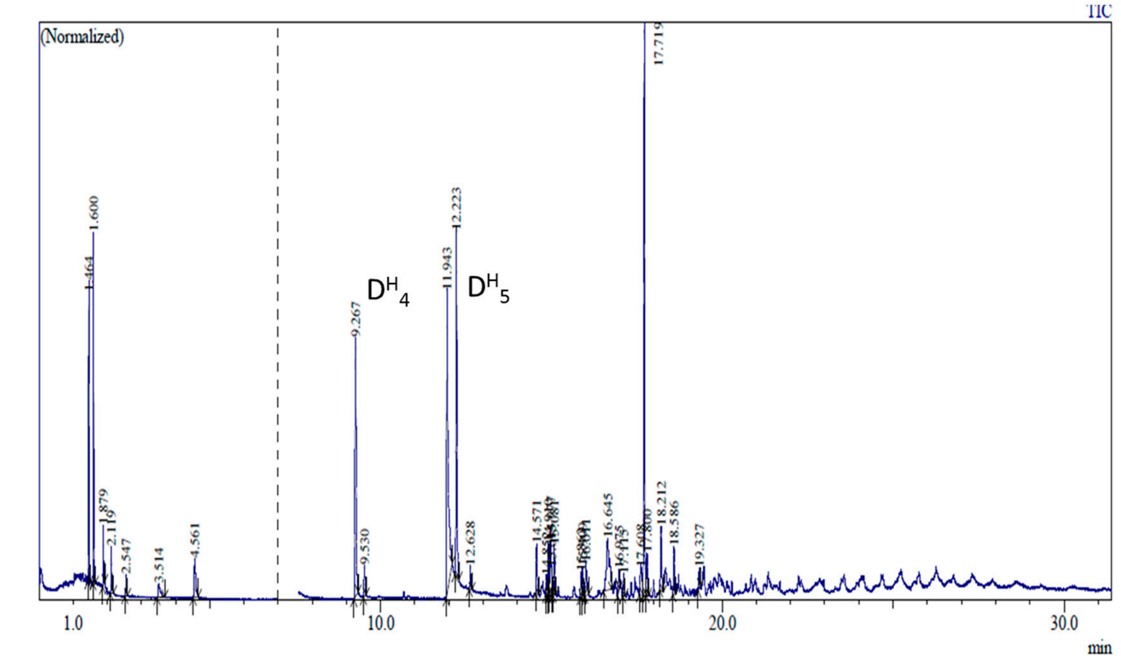

Figure S3A. GCMS chromatogram of the reaction mixture of 20 wt % PHMS solution in toluene in the presence of 1x 10^{-2} mol/L of $B(C_6F_5)_3$ at 30 °C

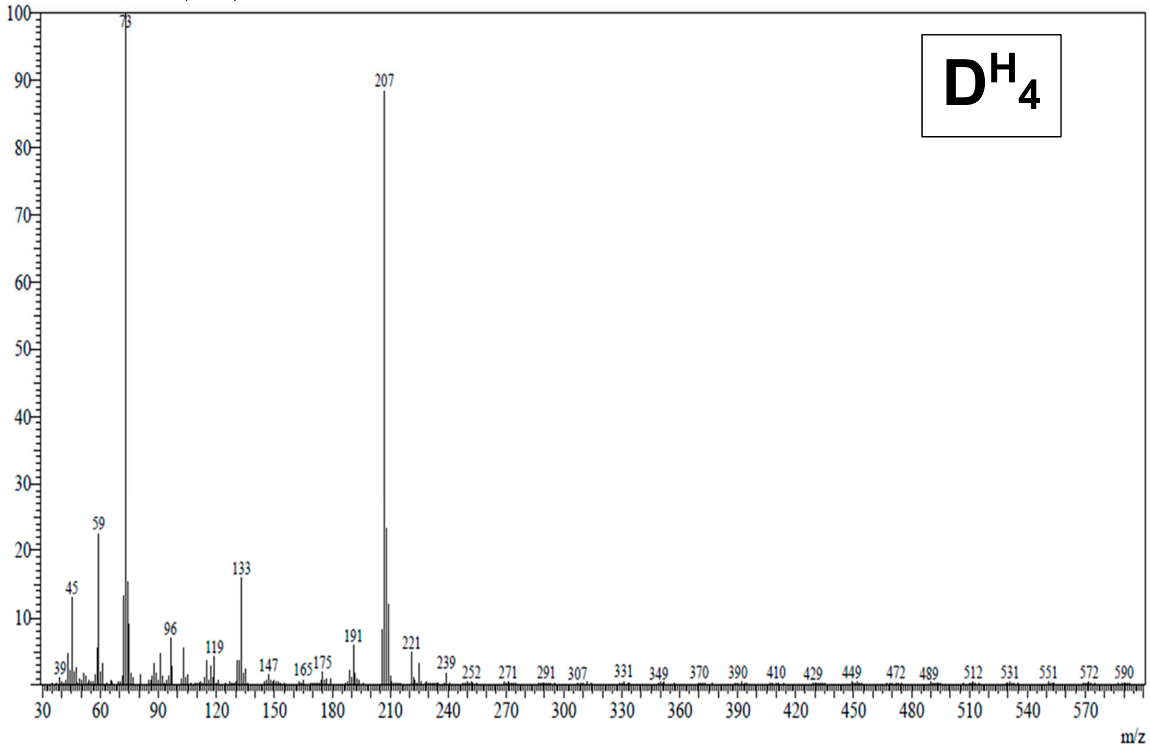

Figure S3B. MS analysis of the peak D^H_4

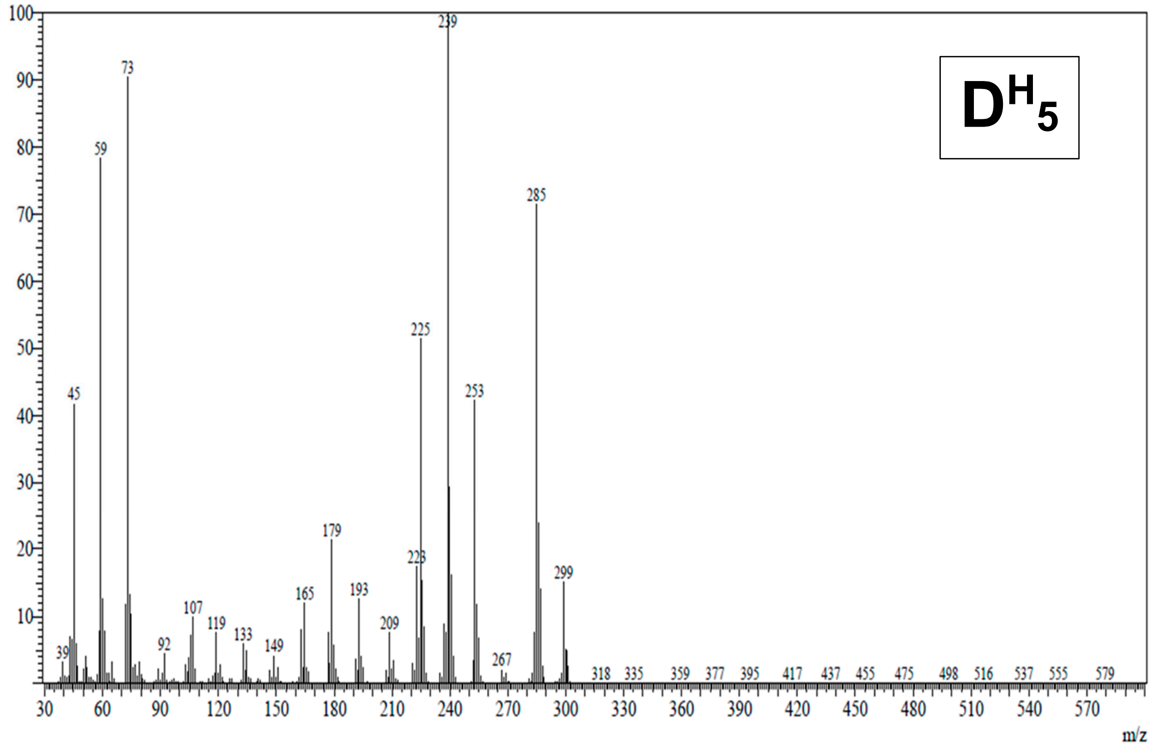

Figure S3C. MS analysis of the peak D^{H}_{5}

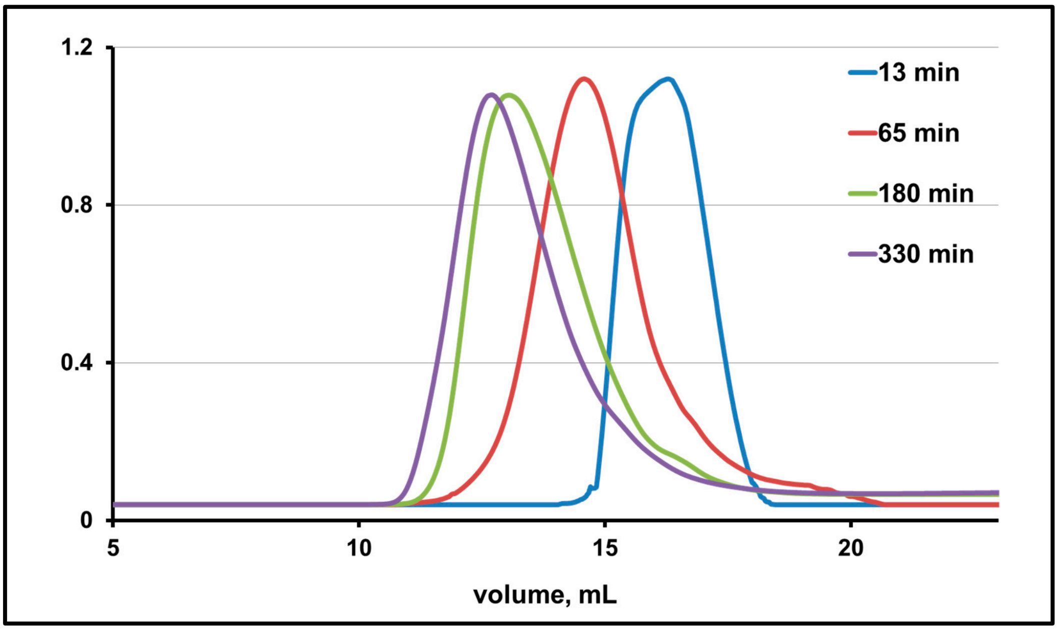

Figure S4. The variation of the molar mass distribution of the PHMS polymer during the restructuring in 20 w% toluene solution at 30 °C in the presence of $1x10^{-2}$ mol/L TPFPB obtained by Multi-Angle Light Scattering detector.

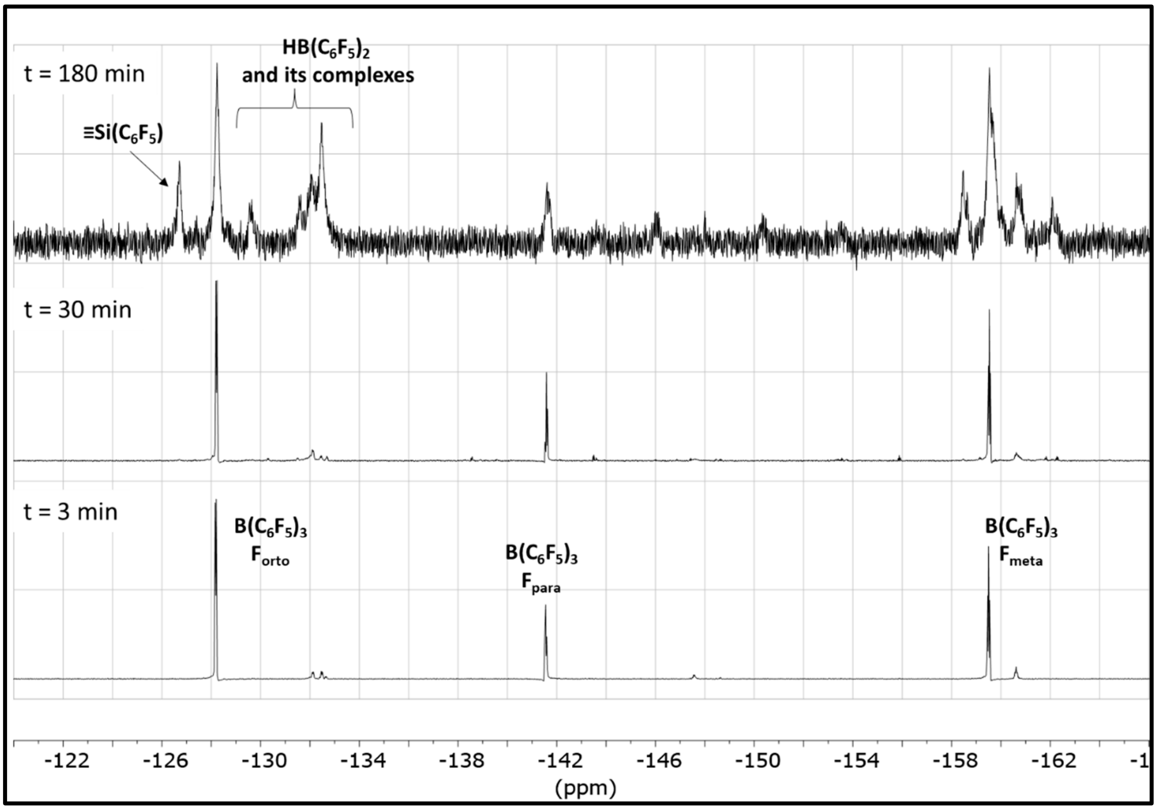

Figure S5. ^{19}F NMR spectra obtained during the reaction of 20 wt % PHMS solution in toluene in the presence of 1 10^{-2} mol/L of $B(C_6F_5)_3$ at 30 °C

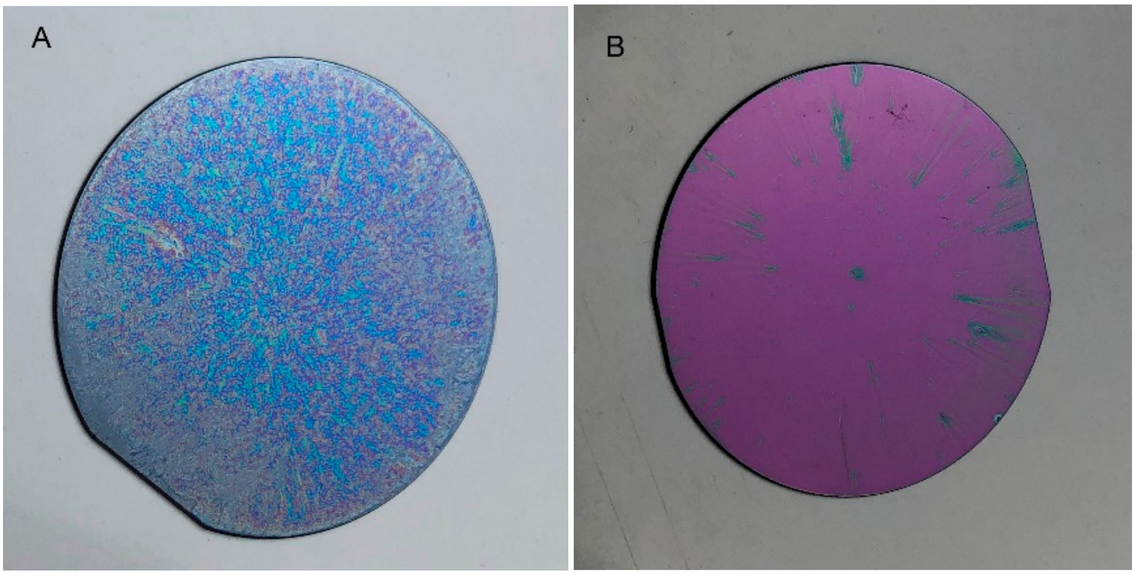

Figure S6. A – image of the poor quality film (Table 3, Film #1); **B** – image of the good quality film (Table 3, Film # 2).

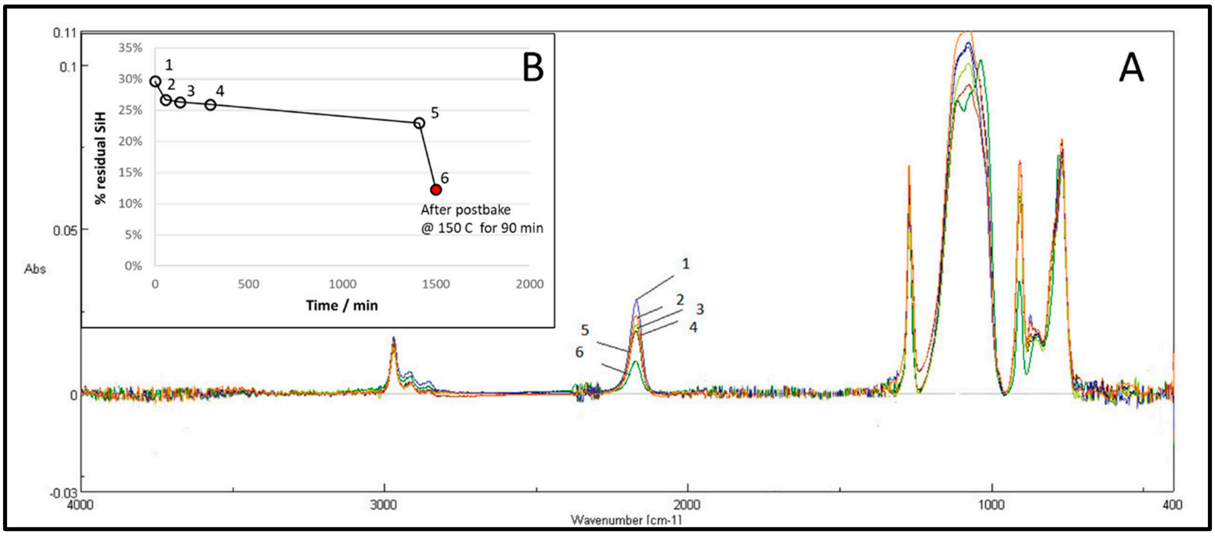

Figure S7. A - IR spectra of the solid film #2 hold at ambient conditions for 24h. **B –** SiH conversion vs time of holding the solid film #2 at ambient conditions and after 90 min postbake at 150 ˚C.
